# Uretero-Colonic Fistula at a Previous Colon Anastomosis Site: A Case Report

**DOI:** 10.7759/cureus.40154

**Published:** 2023-06-08

**Authors:** Kevin D Kunitsky, Chase Cavayero

**Affiliations:** 1 Department of Urology, Kansas City University, Kansas City, USA; 2 Department of Urology, Lee Health, Fort Myers, USA

**Keywords:** urinary tract infection, bacteroides fragilis, fistula-associated infection, radiation-associated fistula, uretero-colonic fistula, ureteral fistulae

## Abstract

Uretero-colonic fistulae are a rare disease resulting from pathologic connection between the ureter and colon, which can be difficult to diagnose. This case report reviews the case of an 83-year-old female with a history of ovarian cancer treated with surgery, radiation, and chemotherapy, who developed a uretero-colonic fistula at a previous colon anastomosis site, which was later diagnosed by ureteroscopy. She was treated with stent placement followed by loop colostomy and was discovered to have metastatic ovarian cancer. She received palliative care consultation and was advised to follow up as an outpatient with the oncology and urology services. Although uretero-colonic fistulae are treatable, treatment depends on patients’ overall clinical picture.

## Introduction

Ureteral fistulae are rare, abnormal connections between the ureter with a neighboring organ, with previous case reports describing uretero-arterial, uretero-cutaneous, and many other types of fistulae [1-3]. Clinical manifestations depend on size and location of the fistula connection, with the most common etiologies being iatrogenic causes, trauma, neoplasms, inflammatory disease, and infection [4]. In this case, we describe our experience with an uretero-colonic fistula at a previous bowel anastomosis site in a patient who previously underwent surgery, chemotherapy, and radiation for ovarian cancer. Although previous cases of uretero-colonic fistula have been reported, there is still limited literature regarding the condition along with difficulty identifying the condition without proceeding with invasive diagnostic techniques [5].

## Case presentation

**Figure 1:**
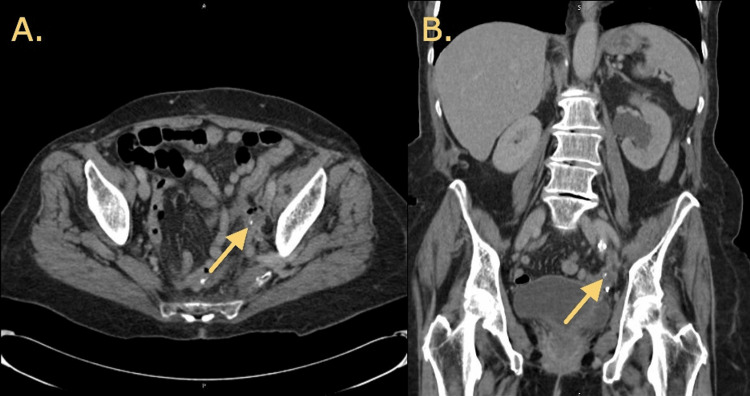
CT imaging demonstrating findings interpreted as a 4-mm obstructing stone. (A) Axial view of CT scan results with arrow indicating region interpreted as a 4-mm stone. (B) Coronal view of CT scan results with arrow indicating region interpreted as a 4-mm stone.

An 83-year-old female with a past medical history of ovarian cancer treated with exploratory laparotomy including bilateral salpingo-oophorectomy, colon resection with sigmoid-sigmoid colonic anastomosis and no obvious surgical complications, chemotherapy, and radiation in 2017 presented to the emergency department with complaints of left-sided upper abdominal pain, back pain, nausea, and vomiting for several days. One week prior to presentation, she had a stent removed, which was initially placed the month prior for hydroureteronephrosis at a different institution. She denied dysuria, hematuria, or obvious changes in urine character. Vital signs demonstrated a blood pressure of 126/57, temperature of 98.2 °F, heart rate of 101 beats per minute, respiratory rate of 19 breaths per minute, and SpO_2_ of 97% on room air. Physical examination including pelvic examination were unremarkable except for left-sided costovertebral angle tenderness. CT imaging revealed a 4-mm obstructing stone in the distal left ureter with mild left-sided hydroureteronephrosis (Figure 1). Urinalysis was negative except for “rare bacteria.” Despite analgesic medications, the patient continued to have intractable pain and was admitted for further evaluation. On day 1 of admission, the patient developed confusion, fever, and chills. Laboratory workup revealed leukocytosis and lactic acidosis. Urine and blood cultures both resulted positive for* Bacteroides fragilis*, raising suspicion for gastrointestinal (GI) source of infection. However, the patient was hesitant to undergo colonoscopy. Urology service opted for emergent left percutaneous nephrostomy tube placement by interventional radiology, and the patient was later transferred to the intensive care unit after developing hypotension.

**Figure 2:**
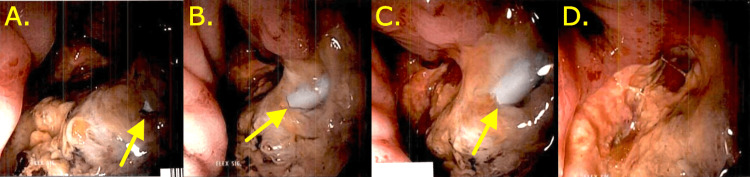
Follow-up flexible sigmoidoscopy images confirming the presence of a fistula. (A-D) Multiple flexible sigmoidoscopy views confirming a uretero-colonic fistula, with arrows indicating ureteral stent placed shortly prior.

Once stabilized, she underwent endoscopic urology evaluation. Cystoscopy findings were unremarkable, and the left ureter was cannulated using a guidewire. Attempts to advance the wire to the collecting system were made; however, the guidewire appeared to turn and enter a lumen significantly medial to the ureter. A ureteroscope was then used to obtain direct visualization. While the distal ureter was normal, 2 cm proximally there was no discernable normal urothelial mucosa, and calcifications on the surgical staple line suspected to be from prior bowel anastomosis were discovered. The guidewire was able to be used to access the collecting system, and an open-ended catheter was advanced over the wire. Retrograde pyelogram confirmed kidney access and the wire was used to place a double-J ureteral stent. Flexible sigmoidoscopy was notable for a large ulceration with significant central necrosis starting from 6 cm into the rectum and extending to 12 cm. There were scattered anastomotic staples as well as exposure of blue tubing, indicative of the ureteral stent and thus confirming the presence of the fistula. (Figure 2).

The patient was evaluated by colorectal surgery and underwent laparoscopic loop colostomy with excision of uretero-colonic fistula and omental mass, histology consistent with metastatic carcinoma with papillary features and psammoma body, likely due to the patient’s known history of ovarian high-grade serous papillary carcinoma. The patient eventually underwent palliative care consultation and was advised to follow up as an outpatient with the oncology and urology services.

## Discussion

The first case of uretero-colonic fistula was described as early as 1956 [6]. Pathogenesis of this disease process is often due to tissue changes in the region of the fistula and, such as in this case, complications relating to prior surgery and radiotherapy. The patient in this report has a history of ovarian cancer treated with surgery requiring colonic anastomosis, chemotherapy, and radiation. Diagnosis of uretero-colonic fistulae is often difficult due to no distinct symptomatology. Without CT imaging demonstrating what was believed to be a stone and leading to further workup, this patient’s fistula may have gone unnoticed as examination findings were nonspecific.

The majority of ureter-associated fistulas are uretero-vaginal fistulas in patients with a history of hysterectomy [7]. In our case, previous radiation and surgery likely caused the prior colonic anastomosis site to fistulize with the patient’s ureter, possibly causing her infectious presentation.

Identifying a fistula on examination can be difficult as there are not many obvious presentations or manifestations of the disease, and identification on imaging is not always clear. Previous cases of uretero-colonic fistula have described symptoms of generalized lower abdominal pain as well as urinary symptoms such as dysuria, both of which were absent in this case [8]. Identifying stool in urine is one possible manifestation for a uretero-colonic fistula and, less likely, possible identification of urine in stool.

Diagnostic evaluation should include CT imaging to evaluate location and size of the fistula; however, it may not always be definitive, as seen in this case. In an ideal scenario, CT imaging can identify fistula, and proceeding to treatment can be expedited. Most notably, urine culture should be obtained, as infectious findings of GI bacteria on culture would strongly support the likelihood of a fistula formation between the GI and genitourinary tracts [9].

Management of uretero-colonic fistula depends on renal function as well as etiology. In cases with poor renal function, nephroureterectomy with excision and closure of the fistula is an option. In cases with good renal function such as this case, stent placement across the fistula followed by surgery is an option. There has also been prior mention of management only using ureteral stent in nonsurgical candidates [10]. Ultimately, management should be aimed at resection of the diseased colon as well as treatment of the underlying cause.

## Conclusions

Uretero-colonic fistulae are rare and can be difficult to diagnose due to various clinical presentations and nondefinitive imaging findings. Associated symptoms are often nonspecific, with many differential diagnoses and symptoms. In this case, direct visualization with a ureteroscope was required to diagnose the fistula, as CT findings were more consistent with what was believed to be a stone as opposed to a fistula, and urinalysis was unremarkable. Importantly, urine and blood cultures were positive for bacteria of GI origin. This was suspicious for some sort of fistula or anatomic abnormality between the ureter and GI tract. Management options often depend on the patient’s renal function and overall clinical picture. In this case, management included ureteral stent placement followed by surgical resection with colostomy.
